# Is Laryngeal Mask a Good Alternative in Children Undergoing Laparoscopic Inguinal Hernia Repair with Percutaneous Internal Ring Suturing Under and Over Two Years Old?

**DOI:** 10.4274/TJAR.2023.221176

**Published:** 2023-06-16

**Authors:** Damla Uysal, Sanem Çakar Turhan, Ergun Ergün, Özlem Selvi Can

**Affiliations:** 1 Department of Anaesthesiology and Reanimation, Ankara University Faculty of Medicine, Ankara, Turkey; 2 Department of Pediatric Surgery, Ankara University Faculty of Medicine, Ankara, Turkey

**Keywords:** Airway management, outpatient anaesthesia, pediatric anaesthesia, perioperative care, pharmacology

## Abstract

**Objective:**

This study aimed to evaluate respiratory parameters during percutaneous internal ring suturing (PIRS) for inguinal hernia repair in two different-aged pediatric patients in whom the airway is provided with a laryngeal mask or endotracheal tube for general anaesthesia.

**Methods:**

After local ethics committee and parental consent, 180 ASAI-II children were randomly allocated to 4 groups; according to their age (0-24 months / 25-144 months) and airway device laryngeal mask (LMA) / endotracheal tube (ETT) used for general anaesthesia (45 children each) for laparoscopic inguinal hernia repair. Standard anaesthesia induction was done with lidocaine, propofol, and fentanyl, and 0.6 mg kg^-1^ rocuronium was added to the ETT groups. Sevoflurane is used for maintenance. Hemodynamic parameters, peak airway pressure, end-tidal carbon dioxide (EtCO_2_), and peripheric oxygen saturation (SpO_2_) values were recorded after induction, before, and during pneumoperitoneum. The duration of anaesthesia, surgery, recovery time, and surgical satisfaction was recorded. Airway complications (cough, laryngospasm, bronchospasm, desaturation, and aspiration) were recorded.

**Results:**

Hundred and eighty patients (45 in each group) were analyzed. Duration of surgery and surgical satisfaction were similar in all groups. Duration of anaesthesia and recovery times were significantly shorter in the LMA groups. Peak airway pressure and EtCO2 levels were significantly lower in the LMA groups. Rare airway complications were observed without significance.

**Conclusion:**

In laparoscopic inguinal hernia repair with the PIRS technique, LMA offered comparable operating conditions and surgical satisfaction.

**Main Points**

• Inguinal hernia repair is one of the pediatric patients’ most frequently applied surgical procedures.

• Open standard herniorrhaphy is a classical technique, but the frequency of laparoscopic techniques is increasing. Percutaneous internal ring suturing (PIRS) for laparoscopic herniorrhaphy in pediatrics enabled the completion of surgery in a relatively short time by using only one umbilical port and needle puncture point.

• The most common technique for airway management during laparoscopy is endotracheal intubation with neuromuscular blocker agent.

• The results of this study revealed that in children aged 0-24 months and 25-144 months, laparoscopic inguinal hernia surgery with PIRS technique can be performed safely with Classic LMA without using muscle relaxants. This way, complications related to muscle relaxants and intubation could be avoided while providing similar surgical conditions and ventilation parameters.

## Introduction

**Figure 1:**
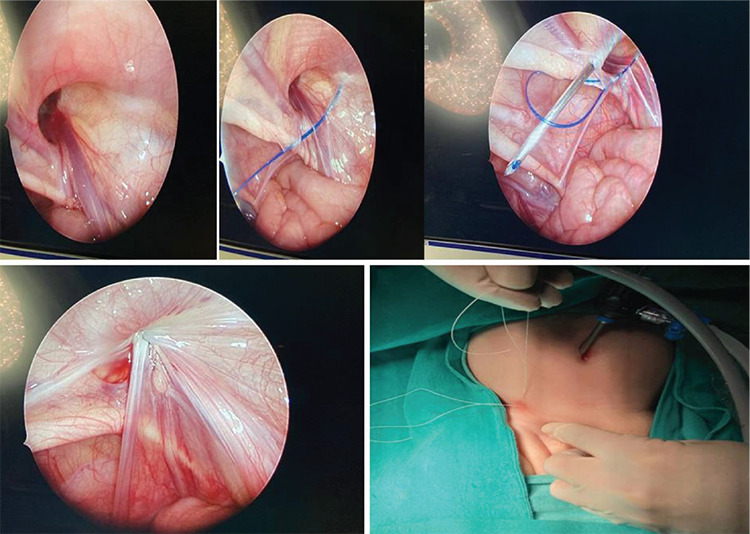
Percutaneous internal ring suturing (PIRS) for laparoscopic herniorrhaphy.

An inguinal hernia is a protrusion of the peritoneum and viscera that needs to be surgically repaired. In pediatric patients, inguinal hernia repair is one of the most frequently applied surgical procedures. While indirect hernia comprises more than 99% of all cases, direct inguinal hernia is rare.^1,2^ Open standard herniorrhaphy is a classical technique, but in recent years there has been an increased use of laparoscopic techniques. Patkowski et al.^3^ described percutaneous internal ring suturing (PIRS) for laparoscopic herniorrhaphy in pediatric surgery, which enabled using only one umbilical port and needle puncture point. It involves the closure of the internal ring extraperitoneal by a needle under laparoscopic guidance. The method is favorable because of the very low risk of recurrence and excellent cosmetic results with a very short surgery with less inflammatory stress (Figure 1).^3,4,5,6,7^ Laparoscopic surgery has another advantage additionally as it gives a chance to diagnose contralateral indirect hernia in the same session.

Laparoscopic surgery has various advantages, including reduced postoperative pain and fewer wound-related complications. The most common technique for airway management during laparoscopy is using a cuffed endotracheal tube (ETT) with a neuromuscular blocker agent and positive pressure ventilation.^8^ With this approach, the risk of aspiration is reduced while providing effective ventilation. Laryngeal mask airway (LMA) is generally used without neuromuscular blocker agent to provide the airway during general anaesthesia. Therefore, in appropriate cases, it can prevent side effects due to muscle relaxants and intubation. Recently, LMA has been used in some laparoscopic surgeries in adults; however, its use in pediatric patients is limited in laparoscopic surgeries.^9,10,11,12,13,14,15,16,17^ Small working spaces in children may even become smaller due to gastrointestinal distention because LMA ventilation may be the major limitation of its use. To the best of our knowledge, the effect of LMA on laparoscopic surgery in different ages of pediatric patients is not compared with ETT. In this prospective randomized controlled trial, children scheduled for single-sided inguinal hernia repair with the PIRS technique were randomized according to their age (0-24 months or 25-144 months) and airway equipment used for airway management (classic LMA and ETT) to analyze respiratory parameters and operating conditions.

## Methods

This randomized controlled study was conducted prospectively after ethical approval (Ankara University Clinical Research Ethics Committee; 17.06.2021 / I6-402-21) between August 2021 and July 2022 in the operating rooms of Ankara University İbni Sina Hospital. One hundred eighty ASA I-II children aged 0-144 months who were scheduled for operation due to single-sided inguinal hernia with PIRS technique were included in the study after parents signed informed consent. After the patient was taken into the age-appropriate group, laryngeal mask or ETT usage was decided using a computer-generated allocation system, and patients were randomly divided into four groups, 45 patients in each group according to their age and airway management equipment:

Group Y-LMA (Younger LMA group; 0-24 months)

Group O-LMA (Older LMA group; 25-144 months)

Group Y-ETT (Younger ETT group; 0-24 months)

Group O-ETT (Older ETT group; 25-144 months).

Patients who needed emergency surgery for strangulated or incarcerated inguinal hernia, patients with an anticipated difficult airway, cardiovascular or pulmonary disease, ASA physical status ≥ III, allergy to study drugs, and those who underwent different surgery in addition to hernia repair were excluded from the study. Standard American Society of Anaesthesiology recommendations for perioperative fasting were used.

All patients underwent routine ASA monitoring, and anaesthesia induction was achieved with a face mask with 8% sevoflurane in O_2_ after transferring to the operating room. “Gentle mask ventilation was performed in all patients to prevent gastric and bowel insufflation.” Following losing consciousness, an intravenous (IV) line was inserted. In all patients, IV lidocaine 1 mg kg^-1^ + propofol 2-3 mg kg^-1^ and fentanyl 1 µg kg^-1^ were administered. In ETT groups, rocuronium bromide 0.6 mg kg^-1^) was added. Before placing LMA or ETT, an appropriate nasogastric (NG) tube was inserted to relieve gas and fluid in the stomach. Proper localization of the NG tube was confirmed by auscultation of the epigastrium and gastric aspiration performed before airway intervention, and the NG tube was secured until the end of surgery.

Appropriate cuffed ETT was inserted, inflating the cuff until minimal leakage with 15-20 cm H_2_O and confirming the correct position of the ETT with capnography and auscultation of the chest. ETT selection was made as follows: For children, 2 years of age and older, 3.5 + (age in years / 4) formula was used. For children 1 to <2 years of age, a 3.5 mm internal diameter cuffed endotracheal tube and a 3.0 mm internal diameter cuffed endotracheal tube were used for children <1 year of age. Additional tubes one size larger or smaller than calculated should also be available.

LMA Classic^TM^ (LMA North America, Inc., San Diego, CA, USA) (C-LMA) is used in all LMA groups. The appropriate size of C-LMA was chosen according to the patient’s weight, and proper positioning of LMA was confirmed by adequate chest rising with no audible leak.

After airway device placement, anaesthesia was maintained with 1-1.5 MAC sevoflurane in O_2_ 40%. Ventilatory settings were initially done with a tidal volume (TV) of 6-8 mL kg^-1^ in 4 L min^-1^ of fresh gas flow, EtCO_2_ levels were closely monitored throughout the entire process, and the respiratory rate was adjusted to keep EtCO_2_ levels in the range of 35-40 mmHg during surgery. Peritoneal insufflation pressure was preset and maintained between 6 and 10 and mm high CO_2_ during surgery. Intraabdominal pressure is also recorded during surgery.

Demographic variables, heart rate (HR), mean arterial blood pressure, peripheric oxygen saturation (SpO_2_), peak airway pressure (P peak), and EtCO_2_ levels were recorded before and after airway device placement, each 5 min intervals after pneumoperitoneum and 1 min after desufflation.

Anaesthesia time (from putting a face mask on the patient’s face until stopping sevoflurane), surgical time (after finishing local anaesthetic infiltration before inserting umbilical port to last skin suturing), recovery time (after completing surgery and stopping sevoflurane inhalation to discharging from the operating room to post-anaesthesia care unit). The surgical team was blinded to airway device selection and neuromuscular blocker agent usage. Surgeons’ evaluation of the surgical field and conditions with a 5-point Likert scale (1: very poor, 2: poor, 3: fair, 4: good, 5: excellent) was questioned at the end of surgery and recorded.

At the end of the operation, IV paracetamol 6-15 mg kg^-1^ was administered according to the patient’s age. In the ETT groups, neuromuscular block was reversed with neostigmine 50-70 µg kg^-1^ + atropine 10 µg kg^-1^. Patients who achieved adequate spontaneous ventilation and reflexes were extubated. In the LMA groups, LMA was removed when the patient achieved adequate ventilation. Laryngospasm, bronchospasm, coughing, breath holding, and desaturation (SpO_2_ <90%) during the recovery period were recorded. Patients who achieved a Modified Aldrete score >9 were discharged to the clinical ward from PACU. Sore throat, nausea, and vomiting were evaluated at the postoperative 2^nd^ hour in children who could communicate and answer questions; in the rest, only vomiting was recorded.

### Statistical Analysis

Data analysis was done in the SPSS for Windows 11.5 (SPSS Inc, Chicago, IL, USA) package program. Descriptive statistics are shown as mean ± standard deviation for variables with normal distribution, median (minimum-maximum) for variables with non-normal distribution, and number of subjects (n) and (%) for nominal variables.

Our primary outcome was the presence of differences between groups in terms of recovery time. A preliminary estimate of a sample size of 45 patients per group of 180 patients was determined with an effect size of 0.25, a power of 0.80, and a margin of error 0.05. The sample size calculation was done with the G*Power 3.1.9.7 program.

The significance of the difference between quantitative variables of groups in the study was investigated using the Student-t test/Mann-Whitney U test. Nominal variables were evaluated using the Pearson chi-square/Fisher's exact test. Mixed Design ANOVA was used to determine whether there was a difference between the groups in terms of P peak, intra-abdominal pressure, SpO_2_, and blood pressure values taken from individuals at 5 different time points. *P* < 0.05 was considered statistically significant.

## Results

**Figure 2:**
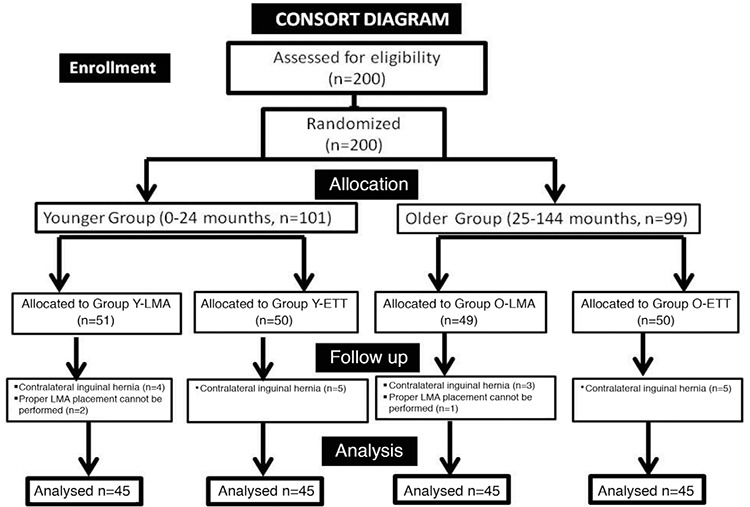
CONSORT flow diagram of patient distribution.

Initially, 200 patients were studied for inclusion in the study. After the patients were randomized into groups, 17 patients were excluded because of contralateral inguinal hernia diagnosis and repair, and three patients were excluded because of improper placement of the LMA. The results of 180 patients, 45 in each of the four groups, were analyzed (Figure 2).

When the results of all 180 patients included in the study were analyzed, 42 were girls (23.3%), and 138 (76.7%) were boys. The mean age of all patients was 39 ± 23.72 months. One hundred and seventy-six patients (97.8%) were ASA I; four (2.2%) were ASA II. The surgical procedure was completed laparoscopically in all patients. The mean duration of anaesthesia for all patients was 43.54 ± 9.01 minutes, the mean operation time was 33.59 ± 9.67 minutes, and the mean recovery time was 9.54 ± 3.17 minutes.

There was no difference in gender, age, weight, height, and ASA physical status between the 0-24 months-old Y-LMA and O-ETT groups. However, the duration of surgery was similar between groups, and anaesthesia and recovery times were significantly higher in the intubated group (*P* < 0.001) (Table 1).

Also, there was no significant difference in gender, age, weight, height, and ASA physical status between the 25-144-month-old Y-LMA and O-ETT groups. Similarly, with younger groups, anaesthesia and recovery times were significantly higher in the intubated group (*P* < 0.001), and the duration of surgery was almost the same (Table 2).

Peak airway pressure difference was significant between the patients in Y-LMA and Y-ETT at all time intervals (*P* < 0.001). At all times, patients in Y-LMA had an average of 3.639 cm H_2_O lower peak airway pressure values than patients aged Y-ETT (*P* < 0.001). In addition, patients in the Y-LMA group had an average of 0.876 mmHg lower EtCO_2_ when compared with patients in the Y-ETT group at all times (*P* < 0.001) (Table 3).

Desaturation was not observed in group Y-LMA and group Y-ETT patients at all time intervals, and there was no significant difference between the groups regarding saturation values. For mean blood pressure, there was a significant difference between the group-independent times and between the groups (*P* < 0.001, and *P*=0.003, respectively). The patients in Group Y-LMA had an average of 2.814 mmHg higher mean blood pressure than patients in Group Y-ETT at all times (*P*=0.026). There was also a significant difference between groups-independent times for the HR and between groups Y-LMA and Y-ETT (*P*=0.002 and *P*=0.003, respectively). Patients in Group Y-LMA had an average of 3.153 units higher HR each time than those in Group Y-ETT. Also, patients in Group Y-LMA had a significant increase in HR in the 1st minute after airway intervention compared with those in Group Y-ETT (*P*=0.018).

Table 4 presents peak airway pressure and EtCO_2_ levels between the groups 25-144 months of age who underwent LMA or ETT. For peak airway pressure, significant differences were found between group-independent times and between groups (*P* < 0.001, and *P* < 0.001, respectively). Patients in Group O-LMA had an average of 2.161 cm H_2_O lower P peak values each time than O-ETT. Also, Group O-LMA had an average of 0.292 mmHg lower EtCO_2_ than patients who underwent ETT at all times (*P* < 0.001) (Table 4). There was no significant difference between the groups regarding SpO_2_ at any time interval.

Desaturation was not observed in Groups O-LMA and O-ETT at all time intervals. For mean blood pressure, patients in O-LMA had an average of 0.550 mmHg higher mean blood pressure than patients in O-ETT at all times, but this difference was not significant (*P*=0.105). There was no significant difference between groups and independent times for the HR between the O-LMA and O-ETT groups (*P*=0.165, and *P*=0.593, respectively).

In the postoperative period, cough was observed in 13 (7.2%) patients during recovery; 4 (2.2%) patients in group Y-LMA, 2 (1.1%) patients in group O-LMA, and group Y-ETT was observed in 3 patients (1.7%), and 4 (2.2%) patients in group O-ETT. During recovery in group Y-LMA, only one patient developed bronchospasm, and one developed desaturation. Two patients in group O-ETT developed laryngospasm, and no blood contamination on the airway device or aspiration was observed in any patients. Nausea and vomiting were not observed in any patient, and sore throat was observed in 5 patients at the second postoperative hour in group O-ETT. The age of the patients or maintaining the airway with LMA or ETT was not found to affect the development of cough, laryngospasm, bronchospasm, or desaturation.

Surgeons’ evaluation of surgical field and conditions using a 5-point Likert scale was 4 points in 6 patients. All the remaining patients were given a score of 5 points, and there was no significant difference in this parameter between general anaesthesia with LMA or ETT in both age groups.

## Discussion

This study showed that using C-LMA in laparoscopic inguinal hernia repair with the PIRS technique in two groups of pediatric patients (0-24 months and 25-144 months) provides similar intraoperative respiratory parameters and surgical conditions with the use of ETT and muscle relaxants. In the LMA groups, surgery times were similar to those in the ETT groups in both ages, but anaesthesia and recovery times were significantly shorter.

In laparoscopic surgeries, intra-abdominal pressure increases due to pneumoperitoneum. Muscle relaxants given during surgery reduce intra-abdominal pressure and help obtain a comfortable working space. Also, increased intra-abdominal pressure decreases lung compliance and may cause increased P peak values.^18^ Our results showed that if the airway is provided with LMA, regardless of age, peak airway pressure was lower in LMA groups compared with ETT groups in both age groups (3.639 and 2.436 cm H_2_O in younger and older groups, respectively). Also, no difference was found between the LMA (without muscle relaxants) and ETT (with muscle relaxants) groups regarding intra-abdominal pressure and ETCO_2_ values in both age groups.

The surgeons may experience difficulties during the procedure due to the lack of muscle relaxation regarding the smaller working area. In this study, the surgical team was blinded to the airway device. Their evaluation and satisfaction were similar between the LMA and ETT groups, and the surgery duration was consistent, indicating that lack of muscle relaxants did not affect the surgical conditions in laparoscopic PIRS surgery. Also, peak airway pressures ​​were below 20 cm H_2_O in all groups, and EtCO_2_ levels could be kept below 40 mmHg during the whole procedure, indicating that LMA without muscle relaxants can be used as an alternative in the younger age group in short-term laparoscopic inguinal hernia operations.

Ozbilgin et al.^12^ conducted a study on adult patients. They placed LMA without muscle relaxants, and ETT was applied using muscle relaxants in laparoscopic gynecological surgeries. In patients who underwent ETT, P peak values ​​were significantly higher at the 2^nd^ minute after intubation and just before extubation. Similar to their results in both age groups, we observed significantly lower airway pressures in the LMA groups. The endotracheal tube, which is preferred for the airway, may have caused an increase in pressure due to the slightly narrowing diameter of the airway compared to supraglottic placed LMA. It may indicate that intubation affecting the infraglottic airway causes more airway reaction than methods affecting the supraglottic airways, such as LMA.

LMA is frequently used in short procedures where general anaesthesia is applied due to its ease of insertion and use without muscle relaxants, less hemodynamic instability during insertion, less metabolic stress response, and a lower risk of tracheal trauma.^9,10,11^ Despite all these, a laryngeal mask is not always suitable for all laparoscopic surgeries, but it can be a good alternative to ETT for short procedures. In appropriate patients and surgical procedures, LMA prevents intubation risks and the adverse effects of residual block due to muscle relaxants. This study achieved good anaesthesia and surgical conditions with LMA for laparoscopic inguinal hernia repair in pediatric patients aged 0-144 months and weighing between 4 and 70 kg. We observed significantly shorter anaesthesia and recovery times in the LMA group in younger patients. Moreover, these results were similar in older patients.

The most important reasons for refraining from using LMA in laparoscopic procedures are the risk of aspiration and inadequate ventilation. We did not observe the aspiration in any group. We believe this is because all interventions were elective, and appropriate fasting periods were achieved. Effective gastric drainage was provided with a nasogastric tube before the airway device was placed in all patients, and there was no need for exaggerated Trendelenburg in the PIRS technique. Ozdamar et al.^19^ compared LMA and ETT on ventilation and gastric pressure in pediatric laparoscopic surgeries, and similar to our results, the nasogastric tube allowed gastric drainage, reduced gastric inflation, and did not affect ventilation. Our study did not use second or third-generation LMAs with gastric drainage channels. This is because the classical LMA is used more frequently than the new generation LMAs in our clinic and general practice for different reasons (such as inaccessibility and lack of appropriate pediatric dimensions). In addition, the gastric drainage tube cannon always be placed correctly and easily through the gastric drainage canal with second or third-generation LMAs, especially in small numbers.^20^

In this study, in all age groups using both ETT and LMA, very few airway complications (cough, laryngospasm, bronchospasm, desaturation) were observed, that did not cause any significant difference between the groups. Common airway complications were observed less in using LMA.^11,17,21,22,23,24^ A laryngeal mask may also provide an advantage in patients with an upper respiratory tract infection (URTI) and who require emergency surgery because of an incarcerated or strangulated inguinal hernia. Also, some children have a frequency of URTI of 6-8 episodes per year, so it may be challenging to schedule the child during a symptom-free interval for elective surgery; LMA will be a better alternative for appropriate surgical procedures in children at high risk of airway complications.^25^

McHoney et al.^26^ has shown that on laparoscopic surgeries, a negative correlation was found between EtCO_2_ value and age, and it was shown that carbon dioxide elimination was higher in young children compared to older age. We did not have any difficulties in any group to keep the target values with close follow-up of EtCO_2_ and ventilation monitoring during laparoscopy.

### Study Limitations

This study has limitations; PIRS is a relatively short surgery and can be performed in a supine or minimal Trendelenburg position. The surgeon’s experience is very influential; if the surgery is prolonged or excessive, Trendelenburg is required, and LMA may not be an appropriate option. Therefore, the results of this study can not be generalized to all laparoscopic surgeries in pediatrics.

## Conclusion

In conclusion, in children aged 0-144 months, laparoscopic inguinal hernia surgery with the PIRS technique can be performed safely with a C-LMA without using muscle relaxants. This way, muscle relaxants, and intubation-related complications can be avoided while providing similar surgical conditions and ventilation parameters.

## Figures and Tables

**Table 1:**
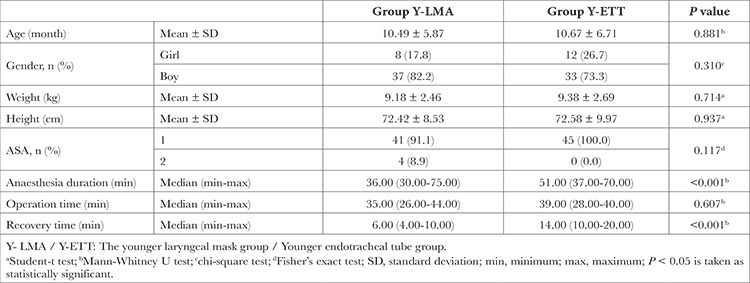
General Descriptors of Patients Aged 0-24 Months in whom LMA or ETT was Used

**Table 2:**
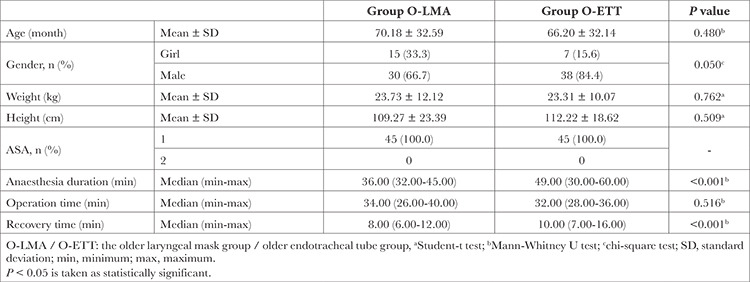
General Descriptors of Patients Aged 25-144 Months in whom LMA or ETT was Used

**Table 3:**
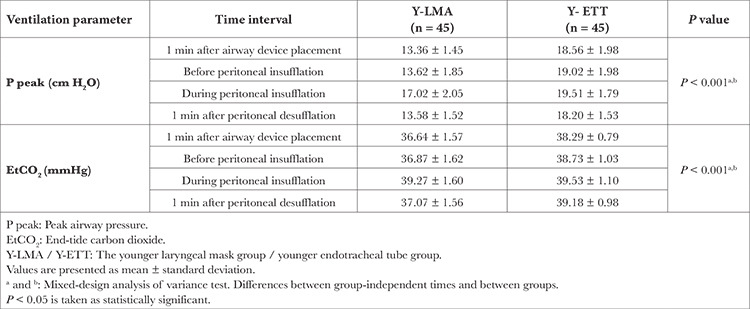
Ventilation Parameters of 0-24 Months Patient Groups During Surgery

**Table 4:**
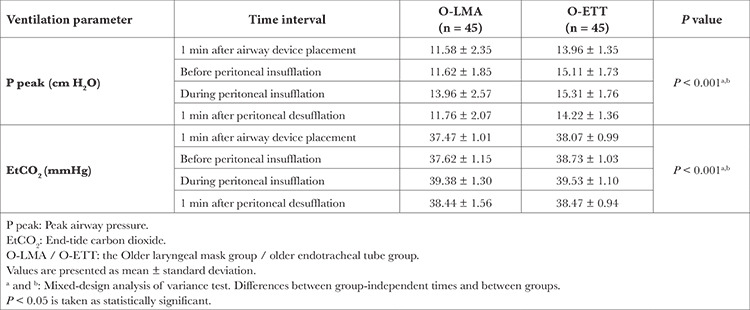
Ventilation Parameters of 25-144 Months Patient Groups During Surgery
